# The Ki-67 Proliferation Index-Related Nomogram to Predict the Response of First-Line Tyrosine Kinase Inhibitors or Chemotherapy in Non-small Cell Lung Cancer Patients With Epidermal Growth Factor Receptor-Mutant Status

**DOI:** 10.3389/fmed.2021.728575

**Published:** 2021-11-05

**Authors:** Weiguo Gu, Mingbin Hu, Linlin Xu, Yuanhui Ren, Jinhong Mei, Weijia Wang, Chunliang Wang

**Affiliations:** ^1^Department of Pathology, The First Affiliated Hospital of Nanchang University, Nanchang, China; ^2^Department of Oncology, The First Affiliated Hospital of Nanchang University, Nanchang, China; ^3^Institute of Molecular Pathology, Nanchang University, Nanchang, China; ^4^Department of Neurosurgery, The First Affiliated Hospital of Nanchang University, Nanchang, China

**Keywords:** non-small cell lung cancer, tyrosine kinase inhibitor, Ki-67, nomogram, survival

## Abstract

**Background:** The correlation between Ki-67 and epidermal growth factor receptor (EGFR)- or Kristen rat sarcoma viral oncogene homolog (KRAS)-mutant status in advanced or postoperative-recurrent non-small cell lung cancer (NSCLC) has fewer studies reported, and the prognostic role of Ki-67 with first-line EGFR-tyrosine kinase inhibitors (TKIs) or chemotherapy remains controversial.

**Methods:** A total of 295 patients were tested for EGFR-mutant status in advanced or postoperative-recurrent NSCLC and received first-line EGFR-TKIs or chemotherapy for treatment. Ki-67 expression was retrospectively analyzed by immunohistochemistry. The Kaplan-Meier method was used to calculate survival rates. The multivariate Cox proportional hazards model was used to generate a nomogram. The established nomogram was validated using the calibration plots.

**Results:** The expression levels of Ki-67 were divided into low (<60%, *n* = 186) and high (≥60%, *n* = 109) groups, based on the receiver operating characteristic curve. The expression levels of Ki-67 were found to be higher in patients with KRAS mutations when compared to KRAS wildtype, and EGFR wildtype was higher than EGFR mutations. The median overall survival (OS) of the low Ki-67 expression group was significantly longer than that of the high Ki-67 group, no matter in all NSCLC, EGFR mutations, EGFR wildtype, KRAS-mutant status, EGFR-TKIs, or chemotherapy of patients (*P* < 0.05). Subgroup analysis showed that the KRAS wildtype or EGFR mutations combine with low Ki-67 expression group had the longest median OS than KRAS mutations or EGFR wildtype combine with Ki-67 high expression group (*P* < 0.05). In the training cohort, the multivariate Cox analysis identified age, serum lactate dehydrogenase (LDH), serum Cyfra211, EGFR mutations, and Ki-67 as independent prognostic factors, and a nomogram was developed based on these covariates. The calibration curve for predicting the 12-, 24-, and 30-month OS showed an optimal agreement between the predicted and actual observed outcomes.

**Conclusions:** The Ki-67 expression-based nomogram can well predict the efficacy of first-line therapy in NSCLC patients with EGFR- or KRAS-mutant status, high expression levels of Ki-67 correlated with a poor prognosis.

## Introduction

Although advances in precision medicine have improved cancer treatment, lung cancer is still the main cause of morbidity and cancer-related mortality worldwide (1, 2). Epidermal growth factor receptor-tyrosine kinase inhibitor (EGFR-TKIs) remains the first-line therapeutic option for advanced non-small cell lung cancer (NSCLC) with EGFR mutation (3, 4). However, after prolonged oral targeted therapy, most patients develop slow or rapidly progressive diseases and drug resistance, such as resistance to osimertinib, a third-generation drug. These drugs fail to bind to original EGFR mutation sites and cause secondary mutations (5), resulting in primary drug resistance (6), secondary drug resistance (7), and activation of bypass pathway (8). It has been shown that tumour, node, metastasis (TNM) stage, tumor differentiation, serum laboratory indexes, or immunohistochemical (IHC) staining may be significant prognostic factors for NSCLC treated with EGFR-TKIs (9, 10).

Ki-67, a proliferative index (PI), is a nuclear marker associated with tumor cell proliferation, it is correlated with progression, metastasis, and prognosis in various malignancies (11). The expression of Ki-67 is an important prognostic indicator for prostate cancer (12), multiple myeloma (13), and breast cancer (14). Moreover, Ki-67 expression is an important biomarker for luminal classification of breast cancer. High expression levels of Ki-67 are associated with neoadjuvant or adjuvant chemotherapy in breast cancer while low expression levels of Ki-67 correlate with the better effect of treatment outcomes (14, 15). Warth et al. (16) investigated the expression levels of Ki-67 in 417 patients with stages I–IV NSCLC, with cutoff values of Ki-67 set at 25% (lung adenocarcinoma, LUAD) and 50% (lung squamous cell carcinoma, LCSC). They found that high expression levels of Ki-67 are associated with worse prognostic outcomes and were, therefore, considered an important prognostic biomarker for stratifying LUAD patients to be administered with adjuvant therapy. Del Gobbo et al. (17) showed that Ki-67 PI and metabolic activities in the LCSC group were higher than those in patients with LUAD with the highest intratumoral heterogeneity in proliferative activities and could also be used as prognostic factors. Zhu et al. (18) investigated the correlation between Ki-67 and EGFR mutations in 523 radically resected NSCLC patients. They found that high expression levels of Ki-67 are associated with poor survival outcomes in both EGFR mutation and wildtype cohorts.

Although Ki-67 is associated with EGFR mutation and poor survival outcomes for NSCLC patients, the relationship between Ki-67 expression and first-line EGFR-TKIs or chemotherapy in NSCLC patients has not been elucidated. Moreover, a limited number of studies have evaluated the association between the KRAS-mutant status and Ki-67. Although the cutoff value of Ki-67 as a prognostic reference in NSCLC has been suggested by a large-scale meta-analysis and several reviews, there is still no consensus on the optimal cutoff value. In this study, we investigated the prognostic value of Ki-67 in NSCLC patients administered with first-line EGFR-TKIs or chemotherapy and established a nomogram for survival prediction.

## Patients and Methods

### Patients and Data Collection

We retrospectively identified patients with histologically confirmed and tested for EGFR mutations in advanced or postoperative-recurrence NSCLC administered with first-line receiving EGFR-TKIs or chemotherapy treatment. This study was performed between January 2014 and March 2019. Approval was obtained by the Ethics Committee of the First Affiliated Hospital of Nanchang University, Jiangxi P.R. China, all patients provided written informed consent before received EGFR-TKIs or chemotherapy and compliance with the declaration of Helsinki. The clinic-pathological characteristics of patients included gender, smoking index, age, pathology, TNM stage, and EGFR mutation subtype (EGFR genetic tests were performed during pre-treatment) among others. The inclusion criteria were (i) all patients were diagnosed with NSCLC by histological or cytological analysis; (ii) patients with EGFR gene mutation were detected and administered with EGFR-TKIs or platinum-containing chemotherapy as first-line therapies; and (iii) enough tissues for Ki-67 IHC staining or Ki-67 had been done in the past. The exclusion criteria were as follows: (i) patients with small-cell lung cancer combined with other malignancies; (ii) patients with unknown EGFR mutation type and incomplete clinical data; (iii) patients with Eastern Cooperative Oncology Group performance status (ECOG PS) score ≥3; and (iv) patients who were intolerant to adverse effects or other comorbidities and those who withdrew from further treatment or lost to follow-up.

### Immunohistochemistry Analysis

Expression levels of Ki-67 in paraffin-embedded tissues from 69 NSCLC patients were detected by IHC, and 226 patients were detected at their first diagnosis. NSCLC tissue sections were sliced from paraffin blocks for IHC analysis of Ki-67 antigens at our cancer pathology center. Antigen retrieval was performed in a water bath using an ethylenediaminetetraacetic acid (EDTA) retrieval buffer (100 ml, Zhongshan Jinqiao company, Beijing). Slides were coated with 50 μl of Ki-67 primary antibody (1:100, 7b11, Zhongshan Jinqiao company, Beijing) and incubated at 4°C refrigerators overnight. Then, they were incubated with a secondary antibody (horseradish peroxidase-labeled mouse/rabbit universal secondary antibody, Dako company, Denmark and Roche company, Switzerland) at 37°C incubators for 15 min. Chromogen diaminobenzidine (DAB Substrate System, Dako, Carpinteria, CA, USA) was used to assess immunoreactivity. Two experienced pathologists, double-blinded to the treatment given, performed histological examinations. Appropriate positive and negative controls were used. Based on the criteria of the NSCLC Working Group, Ki-67 nuclear staining (punctate or diffuse) was found to be positive by selecting the uniformly stained area of tumor cells, and the percentage of Ki-67 positive cells was determined by counting at least 500 tumor cells of immunoreactive nuclei.

### Post-treatment Evaluation Criteria and Follow-Up

Patients were administered with first-line therapy for 3–4 weeks, followed by imaging examination every 2 months and disease evaluation by chest and abdominal CT, bone scan, and craniocerebral nuclear MRI. The Response Evaluation Criteria in Solid Tumors 1.1 (RECIST1.1) was established for short-term effects. Overall survival (OS) was evaluated from the starting date of first-line treatment to the date of death or last follow-up. Follow-up data of NSCLC patients were obtained between January 2014 and March 2019. The cutoff date was July 30, 2020, and the median follow-up period was 25 months.

### Establishment and Validation of the Nomogram

Patients were randomized into the training cohort group (*n* = 206) and validation cohort group (*n* = 89) at a ratio of 2:1 using the R package “rms” analysis. The univariate and multivariate Cox analyses were used to identify the independent prognostic factors in the training cohort and establish a nomogram. The concordance index (C-index) was used to assess the predictive ability of the nomogram, which was internally validated by the Bootstraps method (500 repetitions) for accuracy and unbiased estimates (19, 20). Furthermore, calibration curves of the model for 12-, 24-, 30-month survival outcomes were established to compare the OS outcomes predicted by the nomogram and actual survival outcomes.

### Statistical Analysis

The Ki-67 expression levels were divided into low and high groups by the receiver operating characteristic curve (ROC) (according to the treatment regimens) in all NSCLC patients. Continuous variables of clinical-pathologic factors were converted into categorical variables for analysis. Kaplan-Meier method was used to calculate survival rates while the univariate and multivariate Cox analyses were performed to determine the hazard ratio (HR) and 95% CI for the identification of independent prognostic factors. A *P*-value of <0.05 was considered statistically significant. All statistical analyses were performed using SPSS version 22.0 software (SPSS Inc., Chicago, IL, USA), GraphPad Prism version 5.0 software (Inc., La Jolla, CA, USA), or R statistical software version 4.0.0 (http://www.R-project.org). Cutoff points for peripheral serum LDH and tumor biomarkers were determined as previously described (21).

## Results

### Patient Characteristics

**Figure 1:**
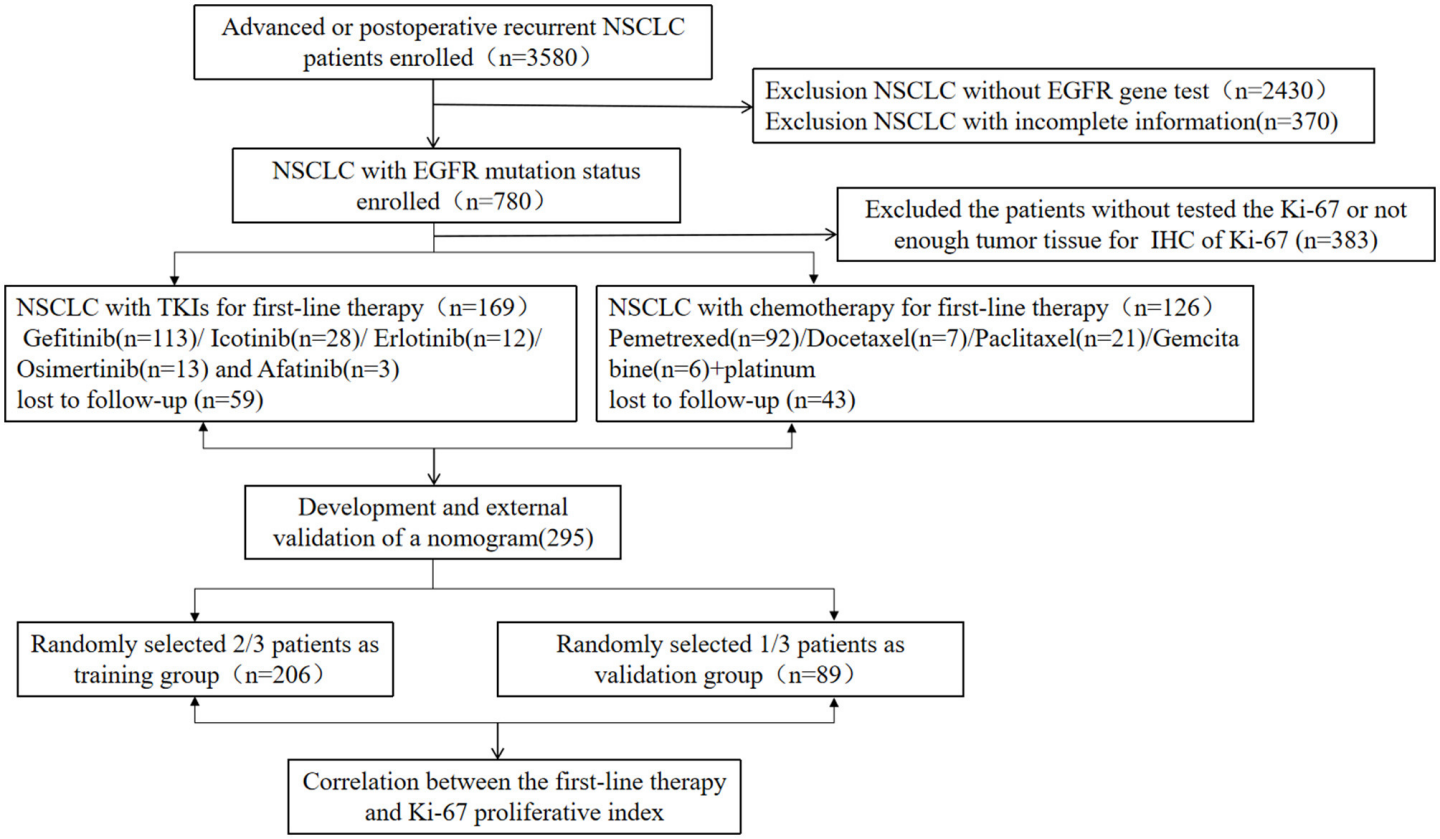
Consort diagram, enrollment, and outcome.

**Figure 2:**
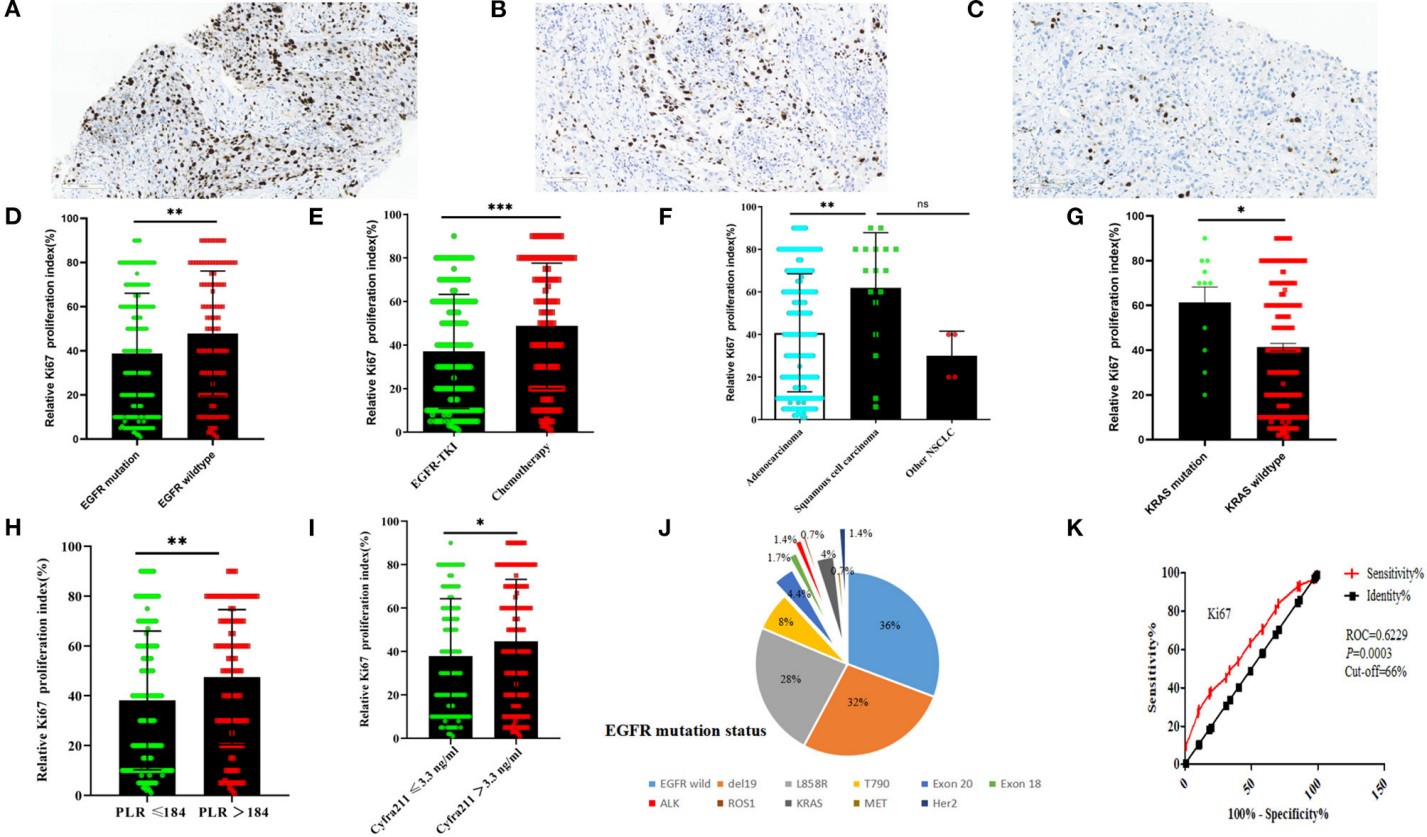
The immunohistochemistry of Ki-67 proliferation index and the relationship between clinical-pathological features in NSCLC. The immunohistochemistry of Ki-67 proliferation index was 85% **(A)**, 50% **(B)**, and 8% **(A)** (scale bar = 100 μm); The EGFR-mutant status **(D)**, treatment regimens **(E)**, pathological types **(F)**, and KRAS-mutant status **(G)**, PLR ratio, **(H)** and serum Cyfra211 **(I)** relationship between Ki-67 proliferation index; **(J)**, the number of patients in NSCLC with EGFR-mutant status; **(K)** the Ki-67 curve of cutoff value (according to the treatment regimens in 295 NSCLC patients). EGFR mutation = del 19/L858R/T790M/Exon18/Exon 20 insertions, PLR = platelet-to-lymphocyte ratio, other NSCLC = adenosquamous carcinoma, large cell carcinoma and unclassified NSCLC. (^ns^*P* > 0.05, ^*^P < 0.05, ^**^P < 0.01, ^***^P < 0.001, mean ± SD, *t*-test). EGFR, epidermal growth factor receptor; NSCLC, non-small cell lung cancer; TKIs, tyrosine kinase inhibitors.

A total of 295 NSCLC patients were included, of which 269 patients had adenocarcinoma, 17 patients had squamous cell carcinoma, two patients had adenosquamous carcinoma, five patients had unclassified NSCLC, while two patients had large cell carcinoma. A flowchart for patient selection in this study is shown in Figure 1. One hundred and fifty-five patients were men while 140 patients were women. The mean age for all patients was 57.5 years (range 22–84 years). There were a total of 188 patients with EGFR mutations (94 patients were positive for del 19, 82 patients for L858R, 25 patients for T790M, five patients for Exon18+, and 13 patients for Exon 20+), 107 patients with EGFR wild-type, 11 patients had KRAS mutations, two patients had Her2 amplifications, two patients had Met amplifications, three patients had EGFR amplifications, four patients had ALK mutations, two patients had ROS1 mutations, five patients had PIK3CA mutations, while three patients had RET mutations (Figure 2J). One hundred and sixty-nine patients who were EGFR-mutant positive were administered with EGFR-TKIs therapy (such as, gefitinib, erlotinib, icotinib, osimertinib, and afatinib) while 126 patients who were EGFR-mutant negative or had EGFR-resistance genes were administered with chemotherapy with platinum-containing regimen or radiotherapy. NSCLC staging at initial diagnosis was based on the American Joint Committee on Cancer (AJCC) staging system. There were 19 patients with unresectable IIIB-IIIC stage, 55 patients with postoperative recurrence, and 221 patients with advanced NSCLC (Table 1).

**Table 1:** Characteristics of 295 patients received first-line therapy in NSCLC with EGFR mutation status.

**Characteristic**		**Ki-67 low group** **(*n* = 186, %)**	**Ki-67 high group** **(*n* = 109, %)**	***P value***
Gender	Male	89 (47.8)	66 (60.6)	0.035
	Female	97 (52.2)	43 (39.4)	
Age (years)	≤ 60	99 (53.2)	57 (52.3)	0.877
	>60	87 (46.8)	52 (47.7)	
ECOG PS	0-1	128 (68.8)	80 (73.4)	0.405
	2-3	58 (31.2)	29 (26.6)	
Smoking index^a^	<400	129 (69.4)	65 (59.6)	0.089
	≥400	57 (30.6)	44 (40.4)	
Pathological type	Adenocarcinoma	175 (94.1)	94 (86.2)	0.022
	Other NSCLC	11 (5.9)	15 (13.8)	
TNM stage	Recurrent	35 (18.8)	20 (18.3)	0.87
	IIIB-C	13 (7.0)	6 (5.5)	
	IV	138 (74.2)	83 (76.1)	
Tumer diameters	≤ 3cm	62 (33.3)	25 (22.9)	0.059
	>3cm	124 (66.7)	84 (77.1)	
Brain metastasis	No	132 (71)	89 (81.7)	0.041
	Yes	54 (29)	20 (18.3)	
EGFR-mutant status	EGFR wildtype	61 (32.8)	46 (42.2)	0.105
	EGFR mutation^d^	125 (67.2)	63 (57.8)	
KRAS mutant	Wildtype	182 (97.8)	102 (93.6)	0.062
	Mutation	4 (2.2)	7 (6.4)	
Treatment regimens	EGFR-TKIs^e^	117 (62.9)	52 (47.7)	0.011
	Chemotherapy^f^	69 (37.1)	57 (52.3)	
PLR ratio^b^	≤ 184	118 (63.4)	52 (47.7)	0.008
	>184	68 (36.6)	57 (52.3)	
NLR ratio^b^	≤ 3.68	124 (66.7)	65 (59.6)	0.224
	>3.68	62 (33.3)	44 (30.4)	
LDH (U/L)^c^	≤ 250	106 (57)	55 (50.5)	0.277
	>250	80 (43)	54 (49.5)	
CEA (ng/ml)^c^	≤ 6.5	67 (36)	49 (45)	0.13
	>6.5	119 (64)	60 (55)	
Cyfra211 (ng/ml)^**c**^	≤ 3.3	80 (43)	33 (30.3)	0.03
	>3.3	106 (57)	76 (69.7)	

*ECOG PS, Eastern Cooperative Oncology Group performance status; EGFR, Epidermal growth factor receptor; PLR, platelet-to-lymphocyte ratio; NLR, neutrophil-to-lymphocyte ratio; LDH, lactate dehydrogenase; CEA, carcinoembryonic antigen; Cyfra211, cytokeratin 19 fragment;Ki-67, nuclear proliferation antigen 67*;

a *number of cigarettes per day × smoking age*,

b *the cut-off points was used mean value*,

c *the cut-off points was used relevant assay kits*.

d *del19/L858/T790M/Exon20/Exon18*,

e *Gefitinib/Erlotinib/Icotinib/Osimertinib/Afatinib*,

f *Pemetrexed/Docetaxel/Paclitaxel/Gemcitabine+platinum or ± radiotherapy*.

### Association Between Ki-67 Expression and Clinic-Pathological Features

Ki-67 was found to be mainly expressed in the nucleus (Figures 2A–C). The Ki-67 expression levels in the EGFR-mutant group were lower than those of the EGFR-wildtype group. Moreover, Ki-67 expression levels in EGFR-TKIs group patients were lower than those of the chemotherapy group (*P* < 0.05, Figures 2D,E). The relationship between pathology and Ki-67 shows that squamous cell carcinoma of Ki-67 was higher than adenocarcinoma (*P* < 0.05, Figure 2F). Ki-67 expression levels in the KRAS-mutant group were higher than those in the KRAS-wildtype group (*P* < 0.05, Figure 2G). Ki-67 expression levels in the low platelet-to-lymphocyte ratio (PLR) group were lower than in the high PLR group (*P* < 0.05, Figure 2H), while The Ki-67 expressions in the low Cyfra211 group were lower than in the high Cyfra211 group (*P* < 0.05, Figure 2I). Moreover, the Ki-67 ROC curve for cutoff points for treatment regimens was established. It was found that the cutoff value of 66% had the highest sensitivity and specificity (sensitivity: 37.3%, specificity: 81.7%, Youden index: 0.19, ROC = 0.6229, *P* = 0.0003; Figure 2K). Therefore, based on Ki-67 expression levels, we divided the patients into low (Ki-67 < 60%, *n* = 186) and high expression groups (Ki-67 ≥ 60%, *n* = 109).

### The Univariate and Multivariate Survival Analyses for First-Line Therapy With Ki-67 in the Primary Cohort

**Figure 3:**
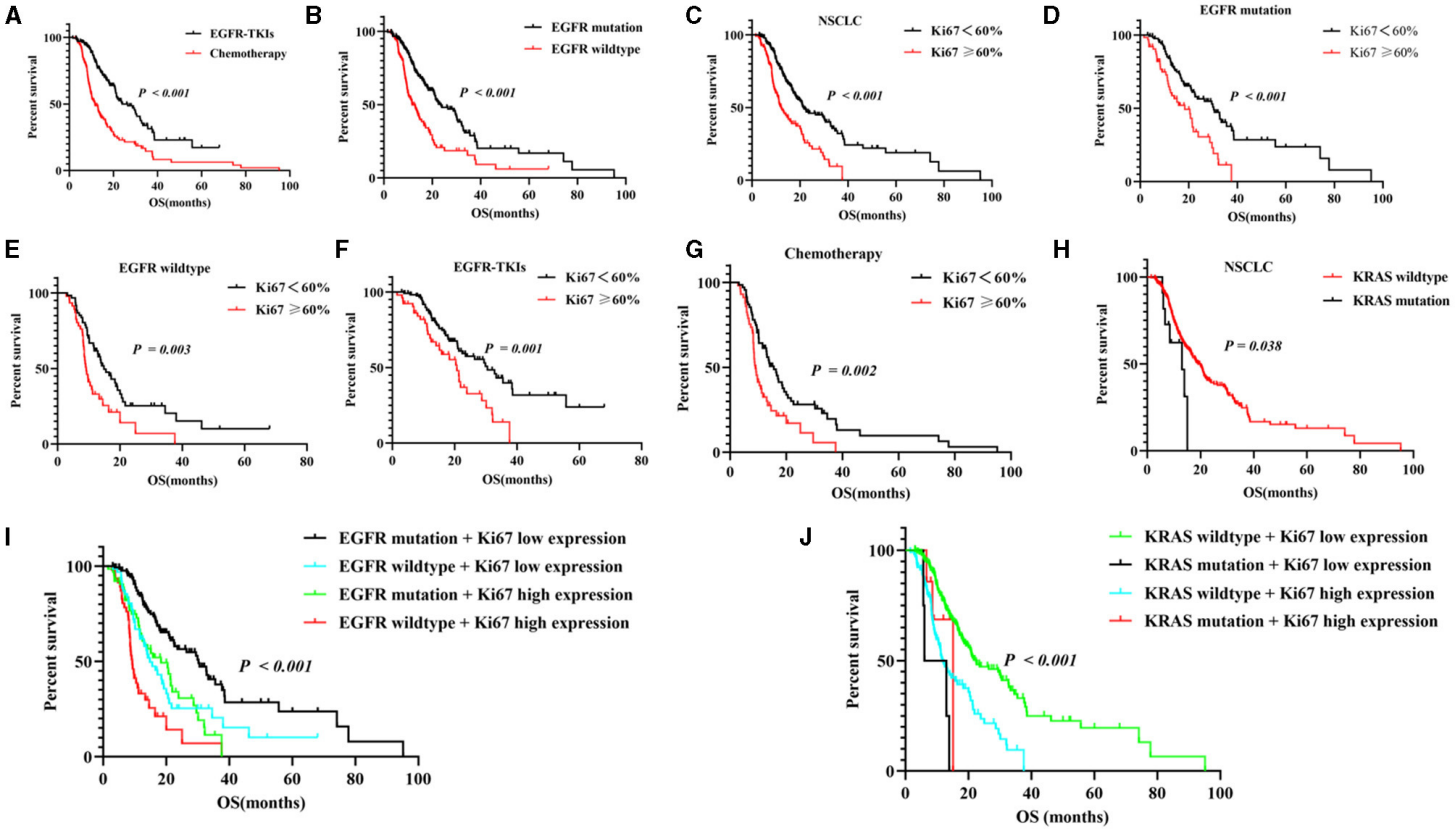
Correlation between NSCLC and Ki-67 proliferation index and first-line therapeutic outcomes in NSCLC patients. **(A)** Compared to EGFR-TKIs and chemotherapy for OS; **(B)** compared to EGFR mutation and EGFR wildtype for OS; **(C)** the two Ki-67 groups and survival curve for first-line treatment in all NSCLC patients; the subgroup analysis of Ki-67 expression levels and survival curve in EGFR mutation **(D)**, EGFR wildtype **(E)**, EGFR-TKIs **(F)** or chemotherapy groups **(G)**; **(H)** the relationship between Ki-67 expression and KRAS-mutant status. **(I,J)** the subgroup analysis of the correlation between Ki-67 expression correlation and EGFR-mutant status or KRAS-mutant status. EGFR-TKIs, epidermal growth factor receptor tyrosine kinase inhibitor, OS, overall survival.

Based on the EGFR-mutant status, the NSCLC patients were assigned to the EGFR-TKIs (*n* = 169) and chemotherapy groups (*n* = 126). The median OS (23.9 vs. 11.6 months, *P* < 0.001, Figure 3A) for the EGFR-TKIs group was longer than for those in the chemotherapy group. In addition, the EGFR mutation group had a longer median OS when compared to the EGFR-wildtype group (23.5 vs. 11.7 months, *P* < 0.001, Figure 3B). Moreover, the low Ki-67 group had a longer median OS compared to the high Ki-67 NSCLC group (21.3 vs. 12.1 months, *P* < 0.001, Figure 3C). Additionally, in EGFR mutation patients, subgroup analysis revealed that the low Ki-67 expression group had a high median OS when compared to the high expression group (30.2 vs. 18.3 months, *P* < 0.001, Figure 3D); in EGFR wildtype patients, the low Ki-67 group had a high median OS than the high expression group (14.8 vs. 9.2 months, *P* < 0.001, Figure 3E). In EGFR-TKIs patients, the median OS for the low Ki-67 group was longer than that of the high Ki-67 group (30.5 vs. 20.7 months, *P* = 0.001, Figure 3F); in chemotherapy patients, the low Ki-67 group had a longer median OS (15.5 vs. 9 months, *P* = 0.001, Figure 3G) than the high group. The KRAS-wildtype group of patients had a longer median OS (19.2 vs. 13 months, *P* = 0.038, Figure 3H) than the KRAS mutation group in all NSCLC patients. Moreover, based on KRAS-mutant status and Ki-67 expression, we found that KRAS wildtype combines with low Ki-67 expression group had the longest median OS (22.4 vs. 6 vs. 15.1 vs. 11.7 months, *P* < 0.001, Figure 3I) than the other three groups; the EGFR mutation group combines with low Ki-67 expression group also had a long median OS (30.2 vs. 14.8 vs. 18.3 vs. 9.2 months, *P* < 0.001, Figure 3J) when compared to the other three groups (Table 2).

**Table 2:** Univariate analysis of primary cohort and training cohort for first-line treatment in NSCLC.

**Characteristic**		**Primary cohort (mOS)**				**Training cohort (mOS)**			
		**N**	**months**	***P***	**HR (95%CI)**	**N**	**months**	***P***	**HR (95%CI)**
Gender	Male	155	14.1		Ref	116	15		Ref
	Female	140	22.4	0.008	0.665 (0.491–0.9)	90	28.7	0.04	0.683 (0.475–0.982)
Age (years)	≤ 60	156	20		Ref	102	20.9		Ref
	>60	139	18.4	0.54	1.098 (0.814–1.483)	104	16.4	0.036	1.469 (1.025–2.105)
ECOG performance	0–1	208	20		Ref	144	18.7		Ref
	2–3	87	15.8	0.101	1.314 (0.948–1.823)	62	15.8	0.217	1.376 (0.942–2.011)
Smoking index	<400	194	20.9		Ref	130	20.9		Ref
	≥400	101	14.3	0.018	1.448 (1.066–1.967)	76	15.5	0.219	1.256 (0.873–1.807)
Pathological type	Adenocarcinoma	269	20.4		Ref	193	20		Ref
	Other NSCLC	26	11.4	<0.001	2.382 (1.466–3.87)	13	14.5	0.158	1.686 (0.816–3.481)
TNM stage	Recurrent	55	21.3	0.005	Ref	42	21.3	0.003	Ref
	IIIB-C	19	9.8	0.002	2.741 (1.439–5.221)	11	9.8	0.002	3.463 (1.563–7.673)
	IV	221	18.7	0.56	1.127 (0.753–1.687)	153	19.2	0.912	1.027 (0.643–1.639)
Brain metastasis	No	221	20.8		Ref	158	19.2		Ref
	Yes	74	16.6	0.168	1.27 (0.904–1.785)	48	16.3	0.332	1.22 (0.816–1.824)
PLR ratio	≤ 184	170	20.9		Ref	121	21.3		Ref
	>184	125	15.5	0.017	1.445 (1.069–1.953)	85	14	0.012	1.582 (1.104–2.266)
NLR ratio	≤ 3.68	189	21.3		Ref	135	21.4		Ref
	>3.68	106	14.3	0.008	1.507 (1.113–2.041)	71	13.9	0.032	1.489 (1.035–2.142)
LDH (U/L)	≤ 250	178	20.9		Ref	114	25		Ref
	>250	117	16.4	0.016	1.447 (1.072–1.954)	92	16.4	0.002	1.77 (1.224–2.561)
CEA (ng/ml)	≤ 6.5	116	18.7		Ref	81	16.5		Ref
	>6.5	179	18.4	0.562	0.914 (0.673–1.24)	125	20.8	0.368	0.847 (0.59–1.216)
Cyfra211 (ng/ml)	≤ 3.3	112	26.4		Ref	77	32.8		Ref
	>3.3	183	15	<0.001	1.832 (1.33–2.523)	129	15	0.001	1.945 (1.317–2.871)
EGFR-mutant status	EGFR wildtype	107	11.7		Ref	76	12.9		Ref
	EGFR mutation	188	23.5	<0.001	0.469 (0.346–0.635)	130	29.6	<0.001	0.451 (0.314–0.649)
Treatment regimens	EGFR-TKIs	169	23.9		Ref	118	28.7		Ref
	Chemotherapy	126	11.6	<0.001	2.386 (1.764–3.228)	88	13.1	<0.001	1.969 (1.377–2.818)
Ki-67	<60%	186	21.3		Ref	128	30.2		Ref
	≥ 60%	109	12.1	<0.001	2.245 (1.646–3.063)	78	12.4	<0.001	2.451 (1.695–3.545)
KRAS mutant	Wildtype	284	19.2		Ref	199	20		Ref
	Mutation	11	13	0.038	2.254 (1.047–4.851)	7	13	0.02	2.68 (1.166–6.163)
Ki-67+EGFR-TKIs	<60%	117	30.5		Ref				
	≥60%	52	20.7	0.001	2.147 (1.347–3.421)				
Ki-67+Chemotherapy	<60%	69	15.5		Ref				
	≥ 60%	57	9	0.002	1.963 (1.285–2.998)				
Ki-67+EGFR mutant	<60%	125	30.2		Ref				
	≥ 60%	63	18.3	<0.001	2.328 (1.528–3.546)				
Ki-67+EGFR wildtype	<60%	61	14.8		Ref				
	≥ 60%	46	9.2	0.003	2.025 (1.272–3.224)				
Ki-67+KRAS	KRAS^wildtype^ + Ki-67 ^low^	182	22.4	<0.001	Ref				
	KRAS^mutation^ + Ki-67 ^low^	4	6	0.001	5.291 (1.922–14.565)				
	KRAS^mutation^ + Ki-67^high^	7	15.1	0.223	2.055 (0.645–6.55)				
	KRAS^wildtype^ + Ki-67^high^	102	11.7	<0.001	2.352 (1.713–3.228)				
Ki-67+EGFR	EGFR^mutation^ + Ki-67^low^	125	30.2	<0.001	Ref				
	EGFR^wildtype^ + Ki-67^low^	61	14.8	<0.001	2.115 (1.415–3.161)				
	EGFR^mutation^ + Ki-67^high^	63	18.3	<0.001	2.226 (1.47–3.371)				
	EGFR^wildtype^ + Ki-67^high^	46	9.2	<0.001	4.641 (2.991–7.202)				

*ECOG PS, Eastern Cooperative Oncology Group performance status; EGFR, Epidermal growth factor receptor; PLR, platelet-to-lymphocyte ratio; NLR, neutrophil-to-lymphocyte ratio; LDH, lactate dehydrogenase; CEA, carcinoembryonic antigen; Cyfra211, cytokeratin 19 fragment; Ki-67, nuclear proliferation antigen 67; Ref, Reference*.

Moreover, univariate analysis showed that gender, TNM stage, smoking index, treatment regimens, pathological type, EGFR mutation, Kristen rat sarcoma viral oncogene homolog (KRAS) mutation, PLR ratio, neutrophil-to-lymphocyte ratio (NLR) ratio, serum LDH, serum Cyfra211, and Ki-67 were correlated with OS and first-line therapeutic outcomes in NSCLC patients (Table 2). Significant factors from univariate analysis (*P* < 0.05) were included in the multivariate Cox analysis, which showed that the TNM stage, serum LDH, treatment regimens, and Ki-67 were independent prognostic factors for OS (*P* < 0.05; Table 3).

**Table 3:** Multivariate analysis of independent risk factors for first-line therapy in training cohort.

**Variable**	**Group**	**Primary cohort (mOS)**			**Training cohort (mOS)**		
		**HR**	**95%CI**	***P value***	**HR**	**95%CI**	***P value***
TNM stage	Recurrent	Ref		0.025			
	IIIB-C	2.308	1.185–4.497	0.014			
	IV	1.061	0.705–1.598	0.775			
Treatment Regimens	EGFR-TKIs	Ref					
	Chemotherapy	2.343	1.722–3.188	<0.001			
Age (years)	≤ 60				Ref		
	>60				1.572	1.083–2.284	0.017
LDH (U/L)	≤ 250	Ref			Ref		
	>250	1.807	1.321–2.471	<0.001	1.516	1.029–2.234	0.035
Cyfra211 (ng/ml)	≤ 3.3				Ref		
	>3.3				1.555	1.029–2.35	0.036
Ki-67	<60%	Ref			Ref		<0.001
	≥ 60%	2.277	1.66–3.123	<0.001	2.245	1.545–3.263	<0.001
EGFR-mutant status	EGFR wildtype				Ref		
	EGFR mutation				0.44	0.304–0.638	<0.001

*ECOG PS, Eastern Cooperative Oncology Group performance status; EGFR, Epidermal growth factor receptor; LDH, lactate dehydrogenase; Cyfra211, cytokeratin 19 fragment; Ki-67, nuclear proliferation antigen 67; Ref, Reference*.

### Association Between the Short-Term Therapeutic Effects of First-Line Therapy and Ki-67 Expressions

Disease progression was evaluated through imaging examination, every 2 months after 3–4 weeks of treatment. For all NSCLC patients, the low Ki-67 expression groups had a higher objective remission rate (ORR, 25.1 vs. 3.1%) and disease control rate (DCR, 59.7 vs. 11.7%) than the high Ki-67 expression groups. Additionally, in the EGFR-TKIs group, subgroup analysis revealed that the low Ki-67 expression groups had a significantly higher ORR (33.7 vs. 2.4%) and DCR (73.9 vs. 12.4%) than the high Ki-67 expression group. Furthermore, in the chemotherapy group, the ORR (13.5 vs. 3.9%) and DCR (40.5 vs. 14.3%) for low Ki-67 expression subgroups were significantly longer than those of the high Ki-67 expression subgroups (Table 4).

**Table 4:** The short-term efficacy comparison among EGFR-TKIs and chemotherapy with Ki-67 in NSCLC.

**RECIST**	**First-line EGFR-TKIs**		**First-line chemotherapy**	
	**Ki-67 <60%**	**Ki-67 ≥ 60%**	**Ki-67 <60%**	**Ki-67 ≥ 60%**
CR	2 (1.2%)	0 (0%)	0 (0%)	1 (0.8%)
PR	55 (32.5%)	4 (2.4%)	17 (13.5%)	4 (3.2%)
SD	68 (40.2%)	17 (10.1%)	32 (25.4%)	9 (7.1%)
PD	11 (6.5%)	12 (7.1%)	25 (19.8%)	36 (28.6%)
ORR	57 (33.7%)	4 (2.4%)	17 (13.5%)	5 (3.9%)
DCR	125 (73.9%)	21 (12.4%)	51 (40.5%)	14 (14.3%)

CR, Complete Response; PR, Partial Response; SD, Stable Disease; PD, Progressive Disease; DCR (disease control rate) = (CR + PR + SD)/total cases ^*^ 100%, ORR (objective remission rate) = (CR + PR)/total cases ^*^ 100%. The short-term efficacy observed at 16 weeks.

### The Univariate and Multivariate Survival Analyses of the Association Between First-Line Therapy and Clinic-Pathologic Features in the Training Cohort

Considering the small number of EGFR mutation cases, all NSCLC patients were analyzed by a nomogram prediction model. Advanced or postoperative-recurrent NSCLC patients in the primary cohort were randomized divided into the training cohorts (*n* = 206) and validation cohorts (*n* = 89) at a ratio of 2:1, using the “rms” R package. In addition, the clinic-pathological features and survival of patients were analyzed in the training cohort. Univariate analysis showed that the gender, age, TNM stages, EGFR mutation, KRAS mutation, treatment regimens, PLR ratio, NLR ratio, serum LDH, serum Cyfra211, and Ki-67 were correlated with the OS following first-line therapy (*P* < 0.05; Table 2). Thereafter, all the significant factors from univariate analysis (*P* < 0.05) were included in the multivariate Cox analysis, which showed that age, serum LDH, serum Cyfra211, EGFR mutation, and Ki-67 were independent prognostic factors associated with first-line therapeutic in NSCLC patients (*P* < 0.05; Table 3).

### Development and Internal Validation of the Nomogram

**Figure 4:**
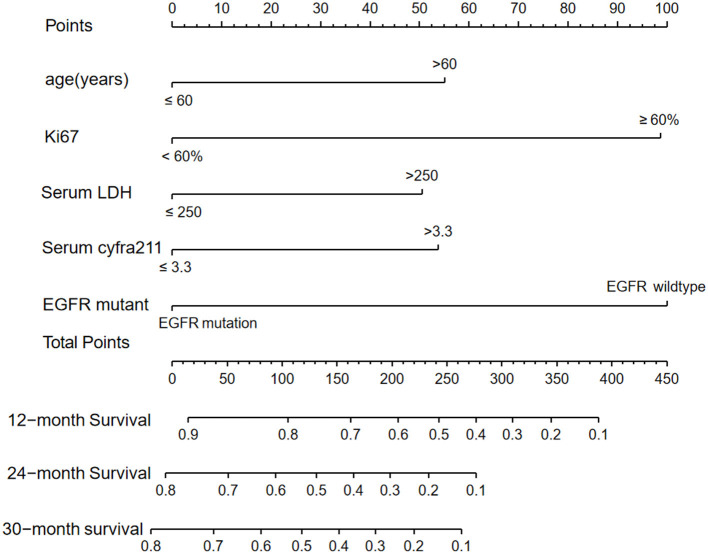
Nomogram predicting the 12-, 24- or 30-month median survival outcomes for NSCLC patients with or without EGFR mutations and treated with first-line therapy. The prediction was done by adding the total points for all the indicators for each variable. For instance, the factor of Ki-67 was vertically downward to the scale labeled “Total Points” axis, and each indicator score was added in total points. Finally, those three factors get the total scores on the “Total Points” axis to the bottom scale and are used to predict the 12-, 24-, and 30-month overall survival probability. EGFR, epidermal growth factor receptor; NSCLC, non-small cell lung cancer.

**Figure 5:**
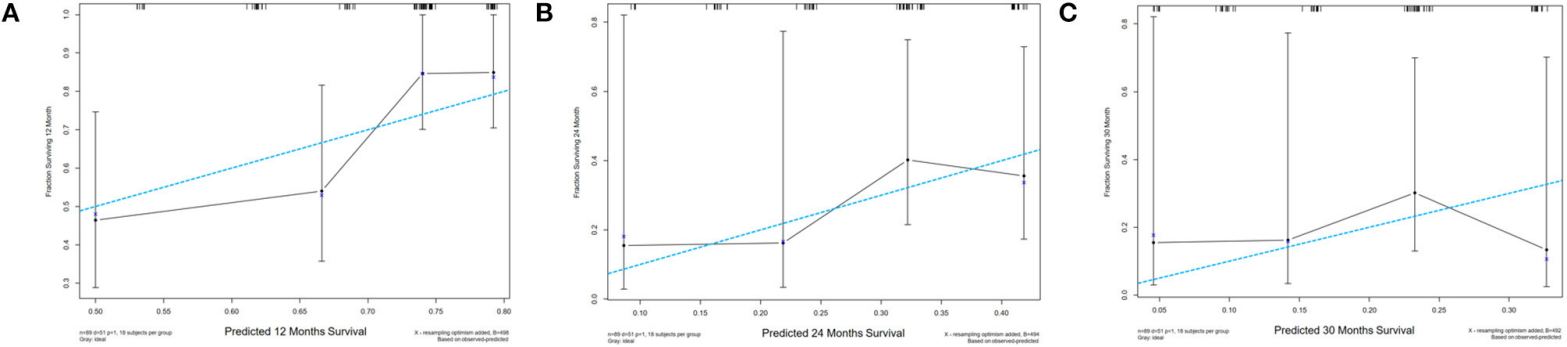
Calibration plots of the nomogram for comparison of the predicted and actual observed overall survival (OS) in **(A)** 12-month, **(B)** 24-month, **(C)** 30-month. The *X*-axis was the predicted OS probability while the *Y*-axis was the observed probability. The vertical bars indicate 95% CIs. The dashed line of dashes indicates a perfect model while the solid line indicates the actual predicted model. OS, overall survival.

The “rms” package in R was used to generate the predictive nomogram through the multivariate Cox analysis in the training cohort. Five variables were incorporated into the nomogram, such as age, serum LDH, serum Cyfra211, EGFR mutation, and Ki-67. The nomogram showed that the EGFR-mutant status and Ki-67 had the most significant contribution to OS among NSCLC patients. Additionally, the total prognostic scores for every variable were calculated and the scores ranged from 0 to 450. Finally, the variables were used to estimate the 12-, 24-, and 30-month OS in NSCLC patients by drawing a straight line through the survival probability scales and obtaining total points (Figure 4). In the internal validation cohort, following first-line therapy, the calibration plots showed that the 12-, 24-, and 30-month OS probabilities predicted by the nomogram were close to the actually observed values in NSCLC (Figures 5A–C).

## Discussion

EGFR-TKIs remain the first-line therapeutic options for advanced NSCLC with EGFR mutations. However, most patients develop slow or rapidly progressive disease and drug resistance after oral-targeted therapy. In addition, nearly 40–50% of the drug-related resistance mechanisms are unknown (5, 6, 22). Previous studies have reported on clinical factors associated with first-line therapy in advanced EGFR positive NSCLC patients. Various nomograms have also been established to predict the survival outcomes for EGFR positive NSCLC patients, and it has been found that IHC biomarkers play an important role in predicting the survival of patients and efficacies of EGFR-TKIs (19, 20, 23–25). However, few studies have evaluated the correlation between Ki-67 expression levels and the first-line therapeutic outcomes in NSCLC patients with either EGFR- or KRAS-mutant status (Table 5).

**Table 5:** Representative studies on risk factors of Ki-67 with EGFR in NSCLC.

**Author, journal, and year of publication**	**Cancers**	**Treatment regimens**	**Risk factors**	**Advantages**	**Limitations**
Ni et al. (26)	postoperative recurrence in completely resected EGFR-mutant NSCLC	adjuvant chemotherapy or radiotherapy without EGFR-TKIs	Tumor size, Ki-67, CK20, and N stage	IHC of Ki-67and CK20 as a new independent risk factor influencing the prognosis of postoperative recurrence NSCLC, was identified and validated. A nomogram was developed and validated	The Ki-67 relationship between EGFR-TKIs and KRAS gene was not detected, and all the patients were postoperative recurrence NSCLC.
Zhu et al. (18)	surgically resected NSCLC patients	Unknown	Former or current smokers, pathological stage and mutant p53-or TTF-1 positive status	High Ki-67 expression was the independent predictor for the worst survival in NSCLC patients with wild-type EGFR	The EGFR-TKIs or chemoradiotherapy with Ki-67 was not analysis, and Ki-67 was not an independent predictor in overall NSCLC group. Not establishment a nomogram model to analyzed the Ki-67 prediction survival of NSCLC
Lin et al. (27)	NSCLC	Unknown	Tumor grade, the slope of the spectral CT	Tumor grade and the slope of the DESCT may be useful for predicting Ki-67 expression and the presence of EGFR mutation in NSCLC	The study only analyzed the DESCT prediction of Ki-67 expression and EGFR genes status, the correlation Ki-67 with EGFR gene were not detceted. The number of case was small and nomograms were not developed and validated
Niemiec et al. (28)	Squamous cell lung cancer (LCSC) patients	Unknown	EGFR labeling index, Ki-67 labeling index	The IHC of EGFR, Ki-67 were independent negative prognostic parameters influencing survivals of LCSC patients	The EGFR gene test was not detected and only by IHC, and the number of case was small. The patients were included only LCSC and were not developed and validated a nomograms

*DESCT, dual-energy spectral computed tomography; NSCLC, advanced non-small cell lung cancer; LCSC, lung squamous cell carcinoma; EGFR-TKIs, Epidermal growth factor receptor tyrosine kinase inhibitor; IHC, immunohistochemistry*.

The present study assessed the clinical-pathological factors and tested the expression of Ki-67 through IHC in advanced or postoperative recurrence NSCLC with EGFR mutations. The study also assessed the relationship between Ki-67 and OS following first-line therapy. Additionally, the multivariate Cox analysis of independent prognostic factors was conducted after which a nomogram was generated to predict the value of Ki-67. To the best of our knowledge, this was the first study to incorporate the expression of Ki-67 and common clinical-pathological factors into a nomogram, to predict survival following first-line treatment in NSCLC with EGFR or KRAS mutations. This may be useful to clinicians in identifying the potential factors affecting first-line therapy.

Immunohistochemical markers are important for the diagnosis and treatment of numerous types of cancers, although the indicators are not fully understood. In addition, Ki-67 has been shown to be expressed in all mitosis in cancer cells and its overexpression increased tumor cell proliferation, invasion, and metastasis of tumors (11, 29–31). In addition, Ki-67 is an important reference index for the diagnosis and treatment of breast, lung, and prostate cancers (12, 14, 16). Notably, Peng et al. (30) assessed the relationship between the epithelial-mesenchymal transition (EMT) phenotype and the proliferative marker, Ki-67, in NSCLC. They found that Ki-67 is closely associated with EMT, and overexpressed Ki-67 is correlated with poor recurrence-free survival (RFS) and OS. The expression levels of Ki-67 have been associated with EGFR mutations and high expression of Ki-67 correlates with poor survival in postoperative NSCLC (18, 32). However, the relationship between first-line EGFR-TKIs or chemotherapy and the EGFR mutation status in NSCLC is unknown. In addition, the cutoff value for Ki-67 as the standard for clinical diagnosis and treatment is still unclear (33).

In this study, two Ki-67 groups were established based on the ROC curve. Expression levels of Ki-67 in the EGFR-positive group were lower than those of the EGFR-wildtype group, and the EGFR-mutant patients administered with EGFR-TKIs had lower expression levels of Ki-67 when compared to the chemotherapy group. Moreover, we assessed the correlation between Ki-67 expression levels and prognostic outcomes in EGFR mutation patients. It was found that in NSCLC, EGFR-TKIs, chemotherapy, EGFR-positive, and EGFR-wildtype groups, the median OS of the low Ki-67 expression group was significantly longer than that of the high Ki-67 expression group. And our findings also revealed that the low Ki-67 expression groups also had a longer ORR and DCR than the high expression category. Furthermore, we interestingly found that EGFR-positive low Ki-67 expression subgroups had the longest median OS time when compared to EGFR-wildtype or EGFR-positive high Ki-67 expression subgroups. Therefore, it is highly likely that Ki-67 is associated with the EGFR mutation status, and first-line treatment regardless of whether the patients are administered with EGFR-TKIs or chemotherapy. Moreover, high expression of Ki-67 was correlated with poor survival, DCR, and ORR, and this may be important in the prognosis of NSCLC patients with either wildtype or mutant EGFR.

The prevalence of LUADs with KRAS mutations in Asia is approximately 10–15% (34), and Johnson et al. (35) showed that the KRAS mutations are associated with poor survival than KRAS wildtype in advanced LUADs. Moreover, the relationship between Ki-67 and KRAS-mutant status in NSCLC, Woo et al. (36) showed that the expression of Ki-67 and the KRAS mutation status is significantly correlated with stage I LUAD. Individuals with higher expressions of Ki-67 and KRAS mutations are at a higher risk of postoperative recurrence than those with low expressions of Ki-67 and without KRAS mutations. Assessment of the correlation between Ki-67 and first-line therapeutic outcomes in advanced or postoperative-recurrent NSCLC patients with KRAS-mutant status, we interestingly found that the Ki-67 expression levels in the KRAS mutation group were higher than KRAS wildtype, and the OS of the KRAS-mutant group was shorter than in the KRAS-wildtype group. Subgroup analysis showed that the KRAS wildtype low Ki-67 expression subgroup had the longest survival time when compared to KRAS mutation low or high Ki-67 expression subgroups. Therefore, KRAS status and Ki-67 expression levels are potential prognostic markers for the prediction of the first-line treatment in advanced or postoperative-recurrent NSCLC.

Nomograms are universally used for predicting cancer risk, and IHC and clinic-pathological data can be used in the assessment of risk factors for predicting tumor survival outcomes. For instance, using the clinicopathological factors available before surgery, Guo et al. (19) built a nomogram for predicting axillary pathologic complete response (pCR) in hormone receptor-positive breast cancer. They found that the nomogram could accurately predict axillary pCR in patients with HR-positive disease. In addition, Keam et al. (37) used clinical features to establish a nomogram for predicting PFS outcomes in NSCLC patients following first-line or other forms of EGFR-TKIs treatment. Their findings showed that performance status, line of chemotherapy, response to EGFR-TKI, and bone metastasis can effectively predict the 6-, 12- and 18-month PFS for NSCLC patients. Most of the studies have focused on the association between clinical factors in NSCLC patients with EGFR mutations and the survival time (20, 21, 26) while few studies have assessed the correlation between Ki-67 and first-line therapeutic outcomes in NSCLC patients with EGFR-mutant status. In this study, 188 EGFR mutant and 107 wildtypes of NSCLC patients who had received either first-line EGFR-TKI or chemotherapy were enrolled. The patients were randomized into the training and validation cohorts at a ratio of 2:1, using the “rms” package in R. The nomogram showed that the EGFR mutation status and Ki-67 had the most significant contribution to OS in NSCLC. The calibration plots also showed that the predicted 12-, 24-, and 30-month OS probabilities were similar to the observed values. Overall, the study showed that the established nomogram, which was based on Ki-67 expression, could effectively predict the efficacy of first-line therapeutic options in NSCLC patients with either wildtype or mutant EGFR.

There are some limitations to this study. First, this was a retrospectively study, and the relationship between Ki-67 expression levels and various adverse reactions was not assessed. In addition, we enrolled 295 NSCLC patients administered with TKIs or chemotherapy, instead of TKIs only, which led to the weakening of the predictive effect of Ki-67 for EGFR-TKIs outcomes. We also included 26 non-adenocarcinoma patients in this study, which limited statistical comparisons with adenocarcinoma patients. Moreover, the Ki-67 ROC curve of the cutoff point was determined using treatment regimens and the levels of sensitivity levels of 37.3% meant that many patients were false negative, which reduced our interest in this prognostic marker in NSCLC patients. Finally, this study was a single-center, small sample size, retrospective study, which may have led to produce selective bias. Therefore, a larger sample size, multi-centered clinical study assessing the association between Ki-67 expression levels and first-line EGFR-TKIs therapeutic outcomes should be performed in NSCLC patients with EGFR mutations. Moreover, the key clinical factors for identifying high-risk patients should be verified in large sample multi-centered studies.

In conclusion, as a proliferation index, the expression levels of Ki-67 are correlated with KRAS- or EGFR mutations and the efficacy of first-line chemotherapy or EGFR-TKIs therapy in advanced or postoperative-recurrent NSCLC. Elevated Ki-67 expression levels are associated with a poor prognosis. The established nomogram based on Ki-67 expression can well predict the efficacy of first-line therapeutic outcomes in NSCLC patients. This is the first study to incorporate Ki-67 expression and built common clinic-pathological factors in the prediction of the survival outcomes after first-line treatment in NSCLC patients with EGFR mutation status and have demonstrated its clinical accuracy, which may help clinical doctors identify potential factors associated with clinical outcomes of first-line therapies or initiate early-intervention treatment regimens for high-risk patient

## Data Availability Statement

The original contributions presented in the study are included in the article/supplementary material, further inquiries can be directed to the corresponding author/s.

## Ethics Statement

This study was approved by the Ethics Institution Committee of First Affiliated Hospital of Nanchang University, Jiangxi P.R. China. All patients provided written informed consent before receiving EGFR-TKIs or chemotherapy and compliance with the declaration of Helsinki was met. All the patients' data are kept confidential.

## Author Contributions

WG and MH were involved in collecting the data and follow-up of patients. WG, YR, LX, and MH were responsible for the conception and design of the study, assisted with the statistical analysis, and wrote the manuscript. CW, WW, and JM contributed to help data analysis and corrected the manuscript. All authors approved the manuscript prior to submission.

## Funding

This study was supported by the National Natural Science Foundation of China (Grant No. 82060517 to LX, 81760448 to CW), the Science and technology project of Jiangxi Health Committee (Grant No. 20203121 to WW), and Science and Technology plan of Jiangxi Administration of Traditional Chinese Medicine (Grant No. 2020B0365). The funding sources had no role in the data collection, analysis, or interpretation.

## Conflict of Interest

The authors declare that the research was conducted in the absence of any commercial or financial relationships that could be construed as a potential conflict of interest.

## Publisher's Note

All claims expressed in this article are solely those of the authors and do not necessarily represent those of their affiliated organizations, or those of the publisher, the editors and the reviewers. Any product that may be evaluated in this article, or claim that may be made by its manufacturer, is not guaranteed or endorsed by the publisher.
